# C/EBP-β Regulates Endoplasmic Reticulum Stress–Triggered Cell Death in Mouse and Human Models

**DOI:** 10.1371/journal.pone.0009516

**Published:** 2010-03-03

**Authors:** Ofir Meir, Efrat Dvash, Ariel Werman, Menachem Rubinstein

**Affiliations:** Department of Molecular Genetics, The Weizmann Institute of Science, Rehovot, Israel; INMI, Italy

## Abstract

Endoplasmic reticulum (ER) stress elicits the unfolded protein response (UPR), initially aimed at coping with the stress, but triggering cell death upon further stress. ER stress induces the C/EBP-® variant Liver-enriched Activating Protein (LAP), followed by the dominant-negative variant, Liver Inhibitory Protein (LIP). However, the distinct role of LAP and LIP in ER stress is unknown. We found that the kinetics of the ER stress-induced expression of LIP overlapped with that of the cell death in mouse B16 melanoma cells. Furthermore, inducible over-expression of LIP augmented ER stress-triggered cell death whereas over-expression of LAP attenuated cell death. Similar results were obtained in human 293T cells. Limited vasculature in tumors triggers hypoxia, nutrient shortage and accumulation of toxic metabolites, all of which eliciting continuous ER stress. We found that LAP promoted and LIP inhibited B16 melanoma tumor progression without affecting angiogenesis or accelerating the cell cycle. Rather, LAP attenuated, whereas LIP augmented tumor ER stress. We therefore suggest that C/EBP-® regulates the transition from the protective to the death–promoting phase of the UPR. We further suggest that the over-expression of LAP observed in many solid tumors promotes tumor progression by attenuating ER stress–triggered tumor cell death.

## Introduction

The endoplasmic reticulum is the site of post-translational processing (refolding, glycosylation and oxidative disulfide bond formation) of secreted and cytoplasmic membrane proteins. The ER is also the site of membrane biogenesis and helps maintain intracellular calcium ion and lipid homeostasis. Accumulation of client proteins or misfolded proteins, overload of free cholesterol, perturbations in calcium ion homeostasis, oxidative stress and xenobiotic toxins rapidly induce ER stress, which triggers an evolutionarily conserved cellular response, termed the Unfolded Protein Response (UPR) [1]. Initial UPR is aimed at coping with the stress by inducing ER chaperones and attenuating general protein translation. Persistent ER stress induces the expression of the C/EBP homologous protein (CHOP), which initiates the cell death machinery [2]. The mechanisms that regulate the transition from the protective phase to the death–promoting phase of the UPR are not known.

ER stress induces the expression of the transcription factor C/EBP-β [3], [4] but there are contradictory reports on the role of C/EBP-β in ER stress. Embryonic fibroblasts from *C/EBP-β*-deficient mice are less sensitive to ER stress–mediated cell death [5], [6]. In contrast, other cell types from these mice exhibit enhanced sensitivity to cell death triggered by growth factor deficiency, exposure to toxins or carcinogens and other stresses [7]–[11]. Other studies found that C/EBP-β is pro-apoptotic [5], [12]–[16]. Because the common C/EBP-β mRNA encodes both transcriptional activators (LAP* and LAP) and a dominant-negative, truncated form (LIP) [17], these opposing observations may be related to the LIP:LAP ratio. Furthermore, the LIP:LAP ratio is not constant, increasing in time, as the ER stress continues [18]. Therefore, further studies are required to determine the specific role of LAP and LIP in the cellular response to ER stress.

ER stress has been associated with many diseases, where it leads to cell death [1], [19]. Because neovascularization lags behind tumor cell proliferation, solid tumors are under continuous ER stress due to nutrient shortage, accumulation of cytotoxic metabolites and hypoxia. However, the cells adapt to this stress through the constitutive activation of the protective phase of the UPR (reviewed in: [2], [20]–[22]). So far, the mechanisms that mediate this adaptation are not fully characterized. The various C/EBP-β isoforms are over-expressed in many types of tumors [8], [23]–[32], but the specific role of LAP and LIP in tumor progression has not been studied. Recently, C/EBP-β was shown to support the survival of neurons under hypoxic conditions, which also trigger ER stress [33]; however, in these studies, both LAP and LIP were either over-expressed or were deleted, and therefore their individual function is not known.

To study the role of C/EBP-β LAP and LIP in ER stress and in tumor progression, we utilized B16 melanoma clones that inducibly over-express the dominant-negative LIP, thereby inhibiting endogenous LAP activity, as well as other clones that inducibly over-express LAP. Our findings suggest that C/EBP-β has a key role in regulating the transition from protective to death promoting UPR; LAP attenuates and LIP augments cell death, and LAP contributes to tumor progression by attenuating ER stress and subsequent cell death.

## Results

### LAP Attenuates and LIP Augments ER Stress-Triggered Cell Death

**Figure 1:**
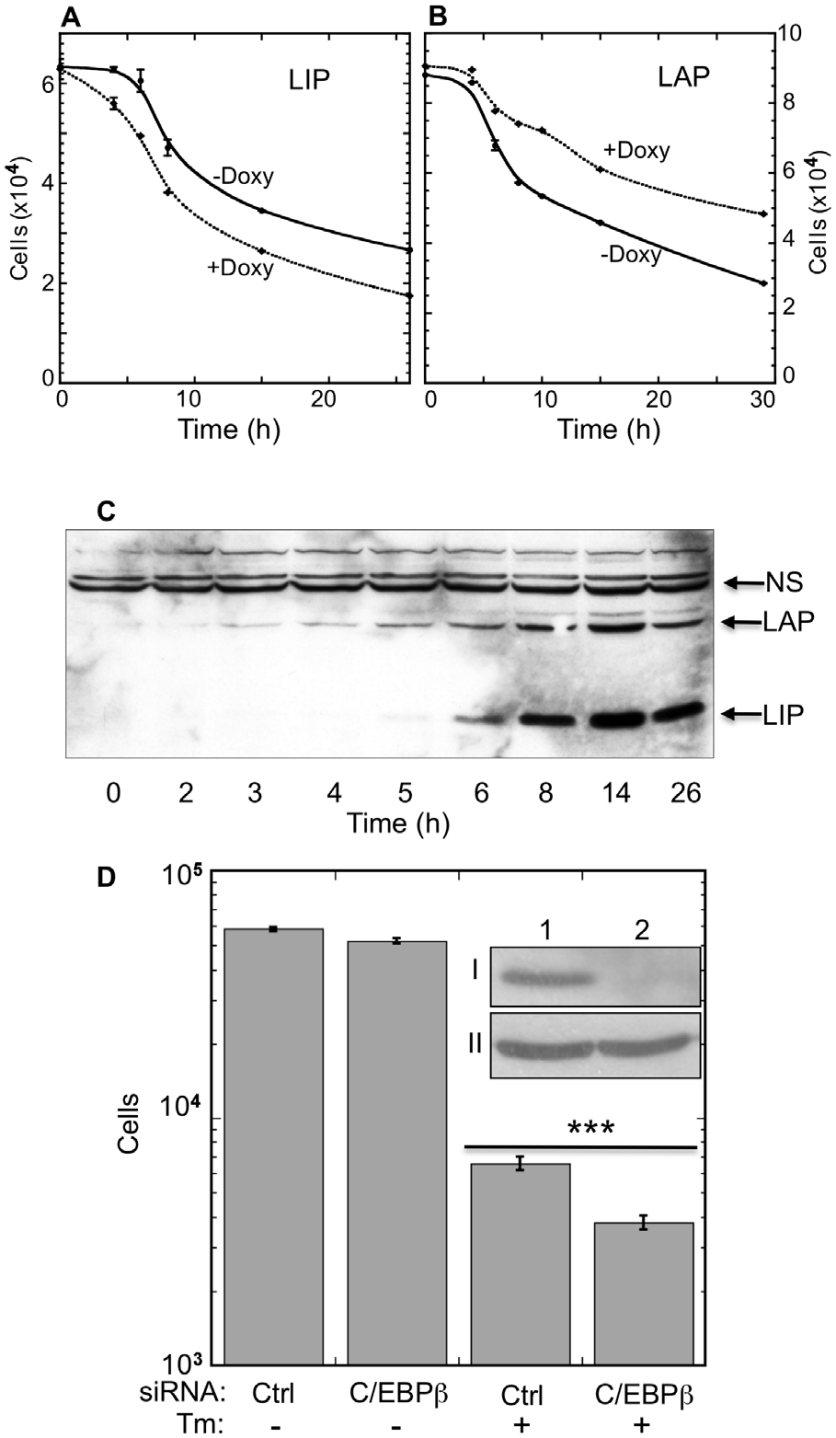
Kinetics and dose-response of ER stress–induced LIP, LAP and cell death. (A) F10.9-4 cells (30,000/well) were cultured in 96-well plates with or without doxycycline (1.2 µg/ml, 24 h). Tunicamycin (0.75 µg/ml) was then added, and the cell number was determined following fixation and crystal violet staining at the indicated times. OD was converted to cell counts using a calibration curve. Data are presented as mean±SD from four replicates. (B) F10.9-3 cells (40,000/well) were cultured and treated with doxycycline and tunicamycin as described in (A). Data are presented as mean±SD from four replicates. (C) F10.9 cells (1.5×10^6^) were seeded in 9 cm plates for 24 h. Tunicamycin was then added to the cells and cell extracts were prepared at the indicated times. Extracts were resolved by SDS-PAGE and immunoblotted with an antibody directed against a common C-terminal peptide of C/EBP-β. NS: non-specific band, serving as a loading control. The blot is a representative of three replicates. (D) F10.9-3 cells were transfected with the indicated siRNA and seeded in 96 well plates (2×10^4^/well). Tunicamycin (Tm) was added after 48 h and viability was determined by Neutral Red staining after 27 h. Data are average±SD of cell counts in 9 replicates. inset: Silencing efficiency as determined by immunoblotting of C/EBP-β LAP (I) vs. actin (II) of cell extracts treated with control siRNA (1) or C/EBP-β siRNA (2).

To study the role of LAP to LIP ratio in ER stress-triggered cell death, we used the specific inducers of ER stress tunicamycin (Tm) and thapsigargin (Tg) and murine B16 melanoma clones that inducibly express either LAP or LIP [34]. Clone F10.9-3 constitutively expresses the Tet repressor and carries a vector with a bidirectional inducible promoter encoding wt LAP and GFP. Immunoblotting with an antibody directed against the common C-terminus of the various C/EBP β isoforms revealed that doxycycline induced only LAP in Clone F10.9-3 (**Figure S1**). Similarly, Clone F10.9-4 expressed only LIP, which is the dominant-negative form of LAP (**Figure S1**). Initially, we triggered ER stress in un-induced cells and compared the progression of cell death and the expression of endogenous LAP and LIP. Cell detachment and death started 4–6 h after addition of tunicamycin, as determined quantitatively by staining of the remaining attached cells with Crystal Violet (**Figures 1A & B** **, solid lines**). Basal LAP and LIP were undetectable by immunoblotting. As previously reported [18], LAP expression was apparent already at 3 h, whereas LIP appeared at 6 h (**Figure 1C**). Thus, initiation and further progression of cell death was temporally associated with endogenous LIP levels.

To further evaluate the role of LAP and LIP in determining the cell fate under ER stress, we studied the impact of their over-expression on the progression of cell death, as determined by Neutral Red staining. Over-expression of LIP in the F10.9-4 cells prior to addition of tunicamycin abolished the 4 h “grace period”, leading to a statistically significant increase of cell death by 4 h (*P*<0.009, N = 4), as well as at all later time points (*P*<0.01, N = 4; **Figure 1A** **, dashed line**). No cell death was seen upon LIP expression in the absence of ER stress (data not shown). Over-expression of LAP had no significant effect in the first 4 h, but significantly attenuated cell death at all other time points (*P*<0.01–0.03; N = 4; **Figure 1B** **, dashed line**). These observations demonstrated that LAP attenuates and LIP augments ER stress-triggered cell death. Since both LAP and LIP were induced past the first 5 h, we evaluated their combined effect on cell viability by siRNA knockdown. Silencing of both LAP and LIP expression significantly augmented the tunicamycin-triggered death of F10.9-3 cells as determined by viability staining with Neutral Red (*P*<0.0001; N = 9; **Figure 1D**). Because LIP lacks a trans-activation domain, it probably augmented cell death by inhibiting ER stress-induced LAP.

**Figure 2:**
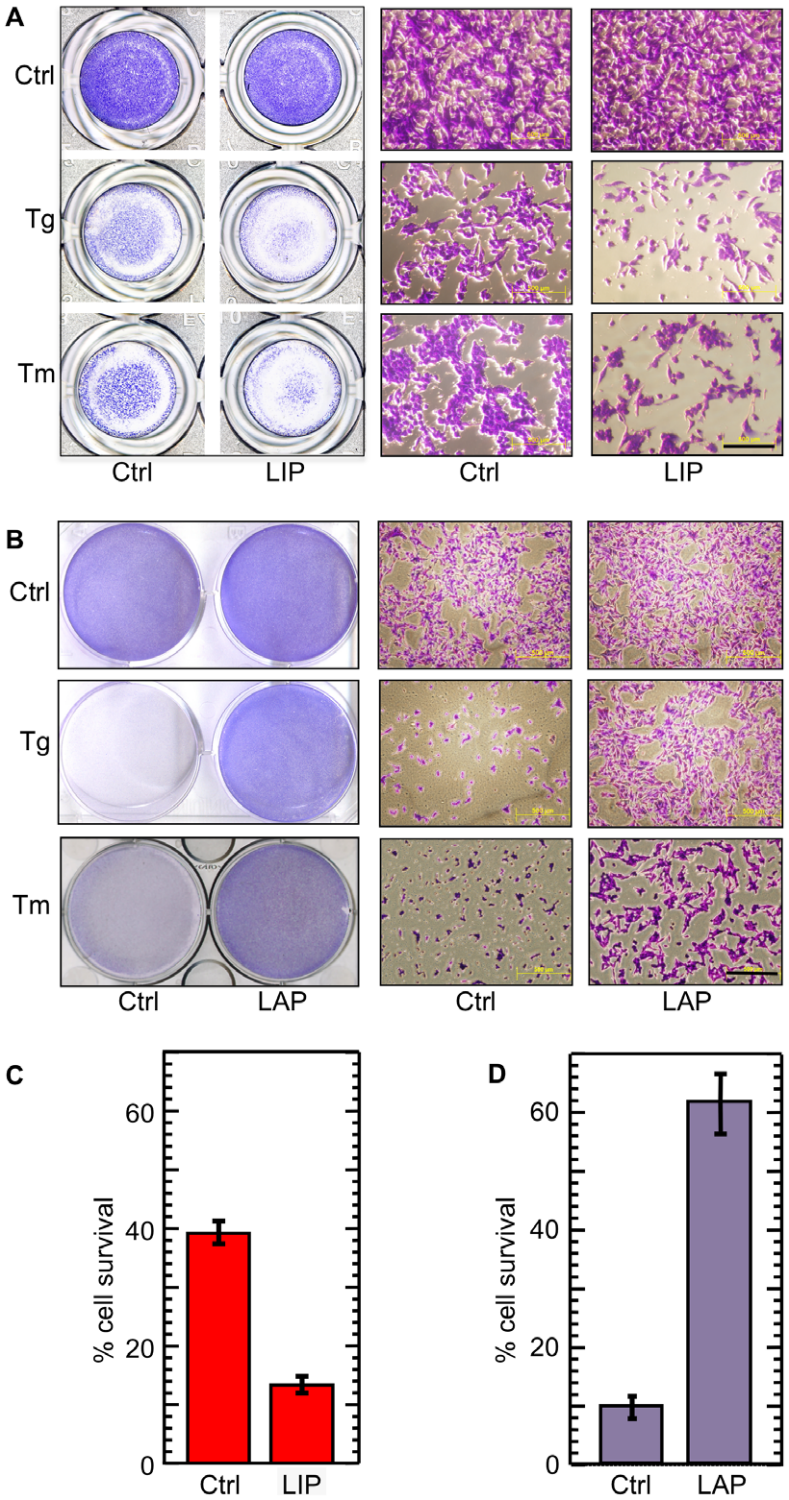
LIP augments and LAP attenuates ER stress–triggered cell death. (A) F10.9-4 cells (2×10^4^/well) were cultured in 96-well plates in media without (Ctrl) or with (LIP) doxycycline. The cells were then treated with diluent (Ctrl), thapsigargin (Tg, 48 h) or tunicamycin (Tm, 24 h). The plates were stained with crystal violet and photographed (left panels, 1x; right panels, light microscopy). The photographs are representative of 5 replicates. Bar = 0.5 mm. (B) F10.9-3 cells (4×10^5^/well) were cultured in 6-well plates without (Ctrl) or with (LAP) doxycycline. Diluent (Ctrl), thapsigargin or tunicamycin were then added. The plates were then stained with crystal violet and photographed as in (A). The photographs are representative of 5 replicates. Bar = 0.5 mm. (C) F10.9-4 cells (1.5×10^4^/well) were cultured in 96-well plates without (Ctrl) or with (LIP) doxycycline. Diluent or tunicamycin were then added. After additional 24 h, the viable cell counts were determined by staining with Neutral Red and the percent survival was calculated. Percent survival was determined on averages of 9 pairs of replicates. (D) F10.9-3 cells (1.5×10^4^/well) were cultured in 96-well plates without (Ctrl) or with (LAP) doxycycline. Diluent or tunicamycin were then added. The cells were stained and percent survival was calculated as in (C).

Microscopic observation of Crystal Violet–stained cultures further demonstrated that LIP over-expression augmented thapsigargin or tunicamycin–triggered cell death at 24 h (**Figure 2A**). In contrast, over-expression of LAP attenuated thapsigargin or tunicamycin–triggered cell death, as determined at 24 and 48 h, respectively (**Figure 2B**). The impact of C/EBP-β on tunicamycin–induced cell death was further determined quantitatively by viability staining with Neutral Red. Over-expression of LIP significantly augmented tunicamycin–induced cell death (*P*<0.0001, N = 9), whereas over-expression of LAP significantly attenuated cell death (*P*<0.0001, N = 9; **Figure 2C, D**, respectively). A similar increase in ER stress-triggered cell death was obtained in three other independent F10.9 LIP-expressing sub-clones following induction with doxycycline. Similarly, ER stress–triggered cell death was attenuated in four other independent F10.9 LAP-expressing sub-clones following doxycycline (data not shown). The enhancement of cell death by LIP was dose–dependent and apparent upon induction with ≥100 ng/ml doxycycline (**Figure S2A**). Similarly, the attenuation of cell death was dose–dependent and apparent upon induction of LAP with ≥100 ng/ml doxycycline (**Figure S2B**).

Microscopic evaluation of Annexin V staining of tunicamycin-challenged cultures revealed lack of apoptotic cells within 24 h of treatment (**Figure S3A**). Rather, tunicamycin triggered cell rounding and detachment from the plate. The detached cells were dead or dying, as determined by propidium iodide staining (**Figure S3B**). Some of the tunicamycin-treated cells remained attached to the plate and although they were propidium iodide negative, they appeared to be dead, as they were condensed and rounded (**Figure S3C**, left panels). Unlike control cells, the LAP–over expressing F10.9-3 cells survived the tunicamycin treatment, they remained attached to the plate and appeared viable (**Figure S3C**, right panels). Furthermore, they propagated normally after removal of the ER stress triggers (data not shown). Based upon these observations we concluded that the ER stress-induced LAP attenuated cell death, whereas LIP augmented cell death, probably by inhibiting or re-directing the pro-survival activity of LAP.

To determine how general is the role of C/EBP-β in ER stress, we studied the impact of C/EBP-β over-expression and knockout in additional cell types. Over-expression of LAP in human 293T cells by transfection with an expression vector designed to encode LAP only (pcLAP, **Figure S4A**) rendered the cells significantly more resistant to tunycamycin-triggered cell death (*P<*0.0007, N = 4, **Figure S4B**). In contrast, over-expression of LIP in human 293T cells by transfection with an expression vector encoding only the LIP open reading frame (pcLIP, **Figure S4A**) rendered the cells significantly more sensitive to tunycamycin-triggered cell death (*P<*0.003, N = 4, **Figure S4B**).

Knockdown of C/EBP-β in human HeLa cells by siRNAs specific to *C/EBP-β* led to efficient silencing of its expression at 48 h, as determined by RT-PCR and immunoblotting with an antibody directed against a common C-terminal peptide of C/EBP-β (**Figure S4C**; left and right panels, respectively). The silencing of C/EBP-β increased the sensitivity of HeLa cells to ER stress–triggered cell death (**Figure S4D**), leading to a statistically significant decrease in the cell counts (*P*<0.0003, N = 4, **Figure S4E**). Over-expression of LAP in human HeLa cells by transfection with pcLAP rendered the cells significantly more resistant to tunicamycin-triggered cell death (*P<*0.001, N = 6, **Figure S4F**). In contrast with all other experiments, over-expression of LIP in HeLa cells by pcLIP also rendered the cells more resistant by 13% to tunicamycin-triggered cell death (*P<*0.002, N = 5). Yet, all other results suggest a general role for C/EBP-β in modulating ER stress–triggered cell death.

### Attenuation of Cell Death by LAP Prevails Over Its Inhibitory Effect on the Cell Cycle

**Figure 3:**
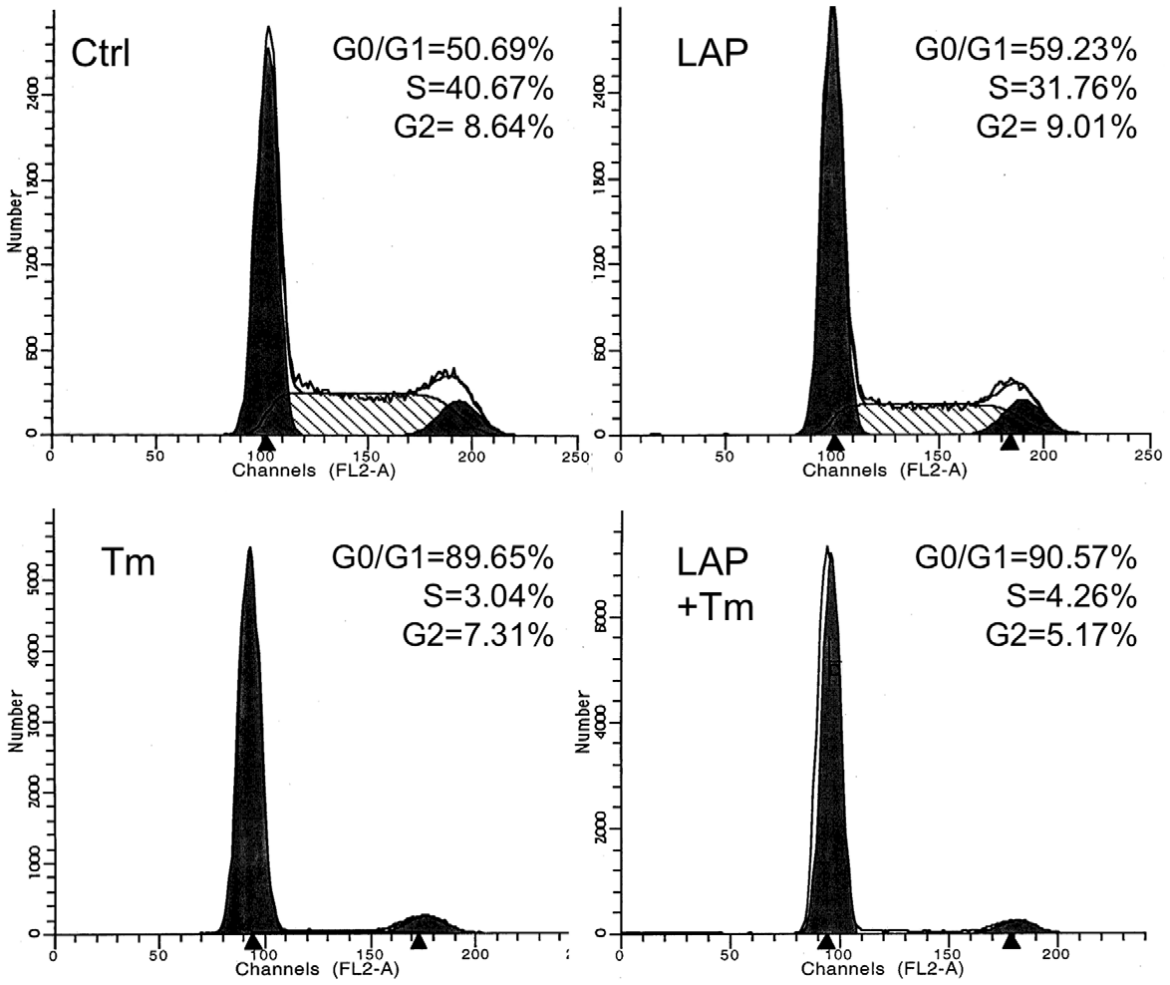
Cell cycle analysis of F10.9-3 cells. F10.9-3 cells (2×10^6^) were cultured in 9 cm plates with or without doxycycline to induce LAP. The cells were then challenged with tunicamycin, collected, permeabilized using 70% ethanol at −20°C, stained with propidium iodide and analyzed using flow cytometry. The graphs are representatives of three replicate experiments.

To study the mechanism by which LAP and LIP regulate the cell fate upon ER stress, we first determined the impact of LAP over-expression on the cell cycle. As previously reported [35], treatment of the B16 melanoma clone F10.9-3 with tunicamycin abolished DNA synthesis, leading to cell cycle arrest, as determined by flow cytometry of propidium iodide–stained cells (**Figure 3** compare the two left panels). Over-expression of LAP in F10.9-3 cells for 24 h prior to treatment with tunicamycin or thapsigargin did not reverse the cell cycle arrest. In fact, LAP lowered the extent of cell proliferation in control cells, reducing the number of cells in S phase by about 25% and increasing the percentage of cells in the G0/G1 phase (**Figure 3**, top panels). The observed LAP-mediated inhibition of cell proliferation was consistent with a previous study on the impact of LAP (LAP-2) on cell cycle progression [26]. Hence, we concluded that the pro-survival activity of LAP must have been the outcome of its effect on cell death rather than on the cell cycle. Furthermore, the attenuation of cell death by LAP under stress prevailed over its inhibitory effect on unstressed cell proliferation. We therefore focused on the impact of LAP and LIP on cell death mechanisms.

### LAP Attenuated Necrotic Cell Death by Lowering CHOP Expression

**Figure 4:**
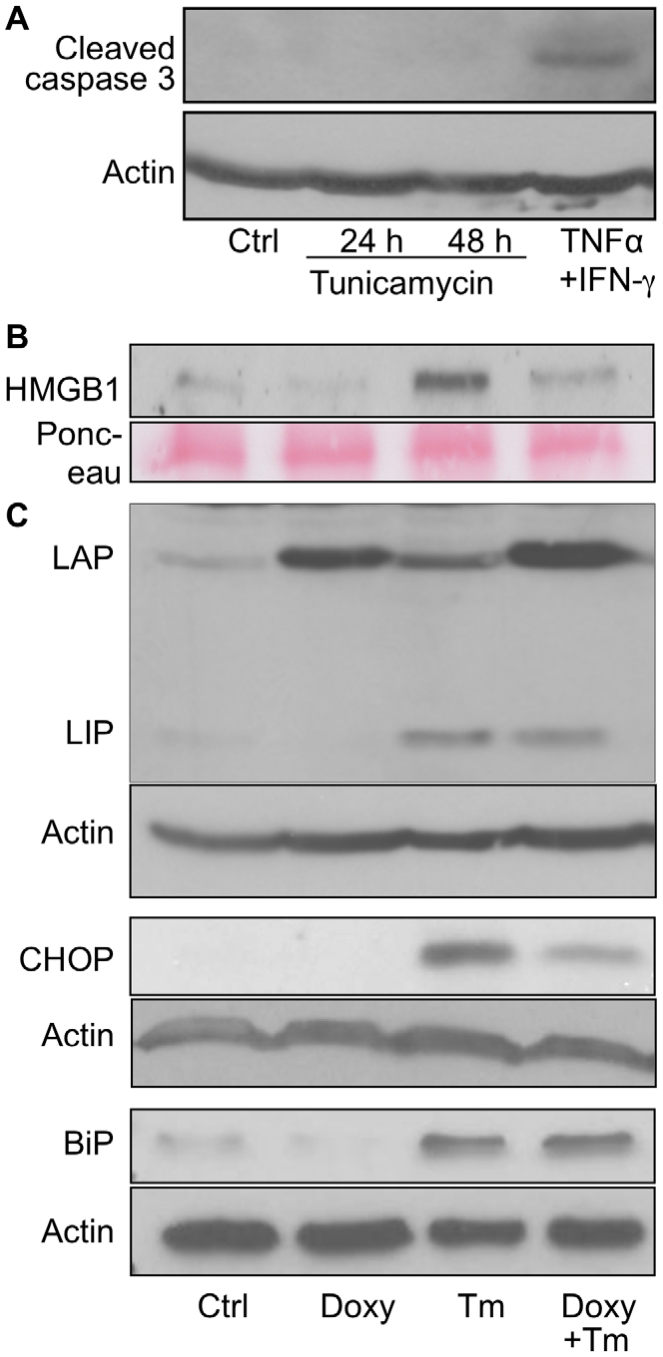
ER stress triggers caspase3–independent cell death, which is attenuated by LAP. (A) F10.9-3 cells (2×10^6^) in 9 cm plates were challenged either with tunicamycin for 24 & 48 h or with murine IFN-γ and TNF-α (1000 IU/ml and 100 ng/ml, respectively, 48 h; used as a positive control for caspase 3-mediated apoptosis). Cell extracts were analyzed by immunoblotting using antibodies to cleaved caspase 3. (B) F10.9-3 cells in 9 cm plates were cultured for 24 h with or without doxycycline (Doxy). The cells were then treated with diluent (Ctrl) or tunicamycin (Tm, 0.25 µg/ml, 24 h). Clarified culture supernatants of the cell cultures (50 µl) were subjected to immunoblotting with antibodies to HMGB1. (C) Total cellular proteins of the cells from (B) were isolated at 24 h and subjected to immunoblot analysis with the indicated antibodies. Each of the immunoblot shown is a representative of three replicate experiments.

Although tunicamycin triggered rounding, detachment and death of the F10.9 cells, we were unable to detect either annexin V-positive cells (**Figure S3A**) or cleaved caspase 3 in the cell extracts (**Figure 4A**). This result was also in accordance with the absence of sub G0 cell population, as revealed by flow cytometry (**Figure 3**). Rather, we found that ER stress triggered the release of High-Mobility Group Box 1 (HMGB1) to the culture supernatant, indicating a mechanism involving necrotic cell death [36]. Furthermore, over-expression of LAP attenuated the release of HMGB1 (**Figure 4B**). Therefore, we concluded that LAP attenuates the ER stress-triggered necrosis of F10.9 cells. The lack of annexin V staining minimizes the possibility that necrosis was initiated by an early stage of a caspase 3-independent apoptotic mechanism. To further study the mechanism by which LAP modulates ER stress, we measured the effect of its over-expression on the early and late ER stress markers Binding Protein (BiP; GRP78) and CHOP in tunicamycin-treated F10.9-3 cells. Immunoblot analysis revealed that over-expression of LAP did not lower the ER stress–induced chaperone BiP. In contrast, LAP reduced the level of the death executor CHOP (**Figure 4C**). These results indicated that LAP does not affect the early protective phase of the UPR. Rather, LAP attenuates the late, death-promoting phase of the UPR.

### LAP Promotes Tumor Progression by Attenuating Cell Death

**Figure 5:**
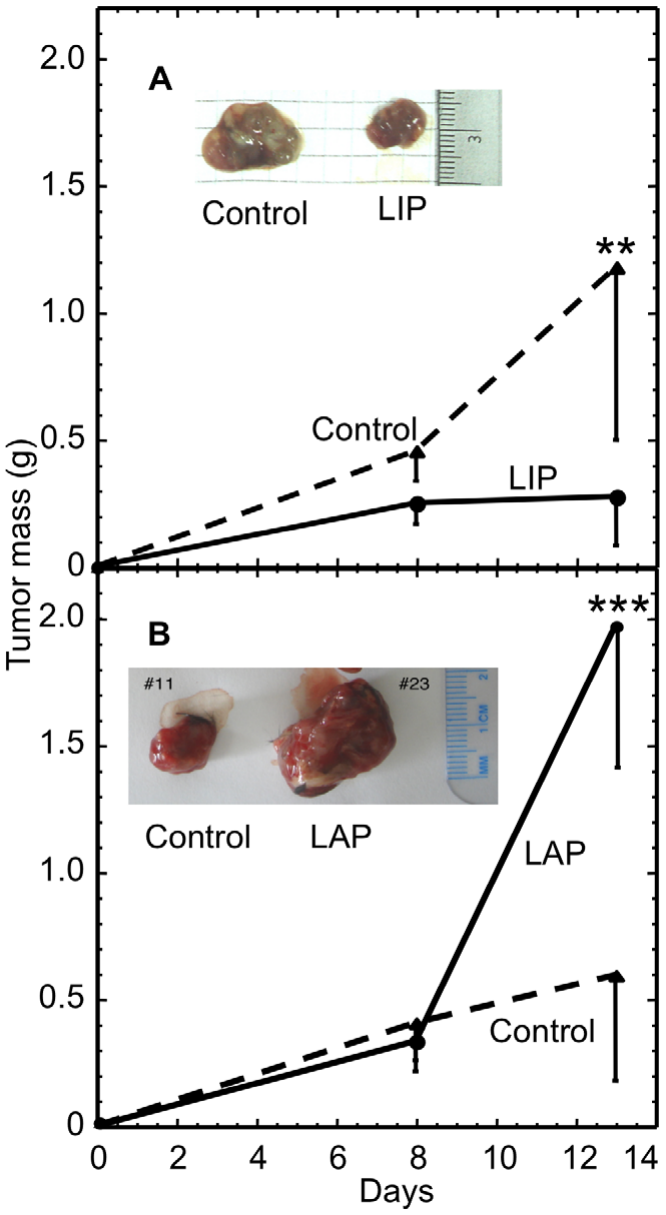
LAP increases B16 melanoma tumor mass. (A) C57/BL6 mice were inoculated with F10.9-4 cells. Dominant negative LIP was induced by doxycycline in the drinking water. Mice were euthanized at day 8 or 13, and the tumors were removed and weighed. Data are presented as the average±SD of 9 tumors. Inset: Photograph of representative tumors. (B) C57/BL6 mice were inoculated with F10.9-3 cells. LAP was induced by doxycycline in the drinking water. Mice were euthanized at day 8 or 13, and the tumors were removed and weighed. Data are presented as the average±SD of 10 tumors. Inset: Photograph of representative tumors.

To study the role of LAP in tumor progression, we inoculated mice subcutaneously either with F10.9-4 cells or with F10.9-3 cells and induced LIP or LAP expression, respectively, by including doxycycline (Doxy; 1 mg/ml) in the drinking water. Palpable tumors were detected in the mice after 8 days and differences in tumor mass were apparent by day 13. Therefore tumors in one group of mice were isolated and examined at day 8 and at day 13 in a second group of mice. The inhibition of endogenous LAP by over-expression of LIP led to a significant attenuation of tumor growth as determined at day 13 (*P*<0.005, N = 9). In contrast, over-expression of LAP significantly increased the tumor mass by fourfold over a 5 day period (*P*<0.001, N = 10; **Figure 5**).

**Figure 6:**
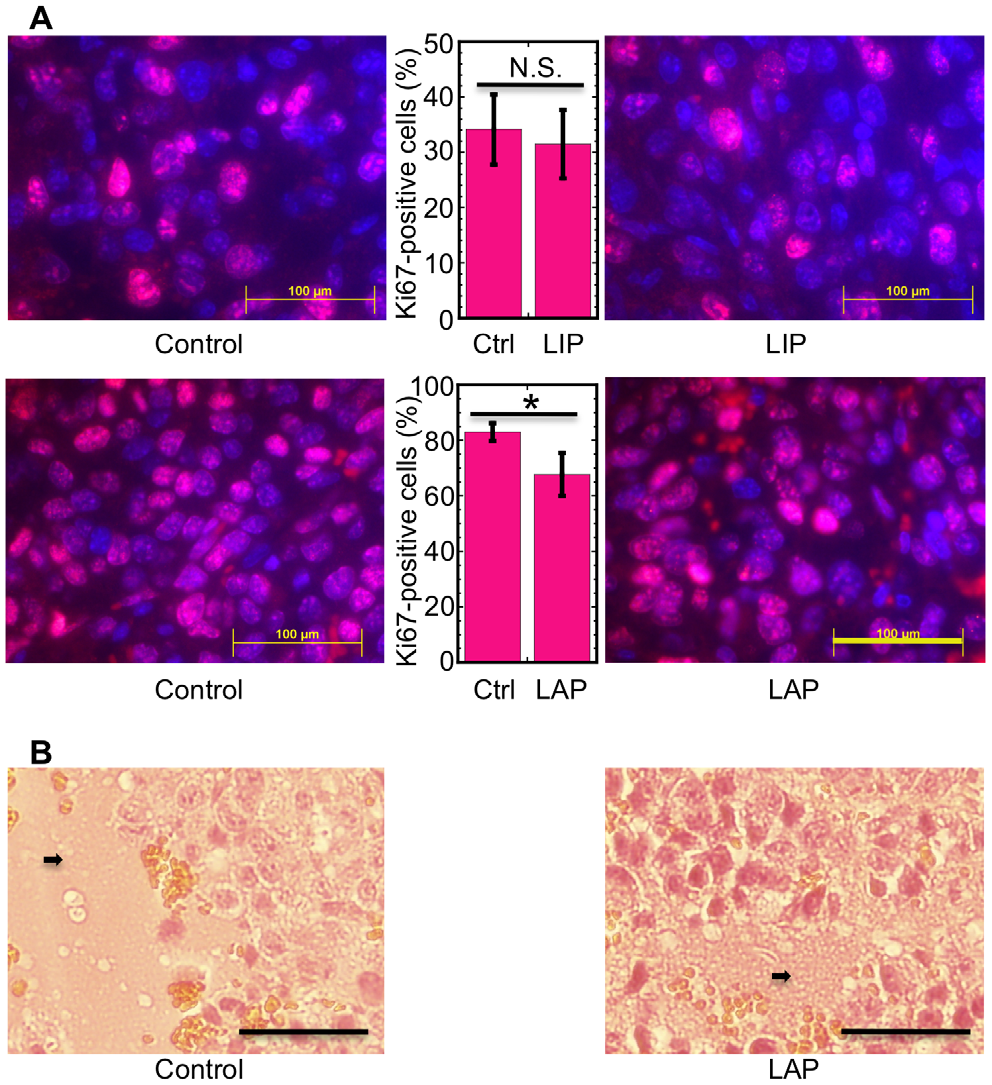
Ki-67 and H&E staining of tumor sections. (A) Sections of control F10.9-4 tumors and dominant negative C/EBP-β LIP–expressing F10.9-4 tumors of similar sizes, isolated at day 8, were immunostained with an antibody against Ki-67, and nuclei were counter-stained with Hoechst 33258 (top left and right panels, respectively). Percent Ki67-positive nuclei were determined by counting sections from 4 control and 4 LIP-expressing tumors (71–94 nuclei/field, middle top panel). Sections of control F10.9-3 tumors and LAP-expressing F10.9-3 tumors of similar sizes, isolated at day 8 (bottom left and right panels, respectively), were prepared and stained as above. Percent Ki67-positive nuclei were determined by counting sections from 4 control and 4 LAP-expressing tumors (60–107 nuclei/field, middle bottom panel). Representative images of sections from the four tumors are shown. Bar = 0.1 mm. (B) Sections of control F10.9-3 tumors and LAP-expressing F10.9-3 tumors of a similar size, isolated at day 8, were stained with hematoxylin and eosin. The block arrows indicate necrotic areas. Bar = 0.1 mm. The photograph is a representative of sections from 5 tumors.

**Figure 7:**
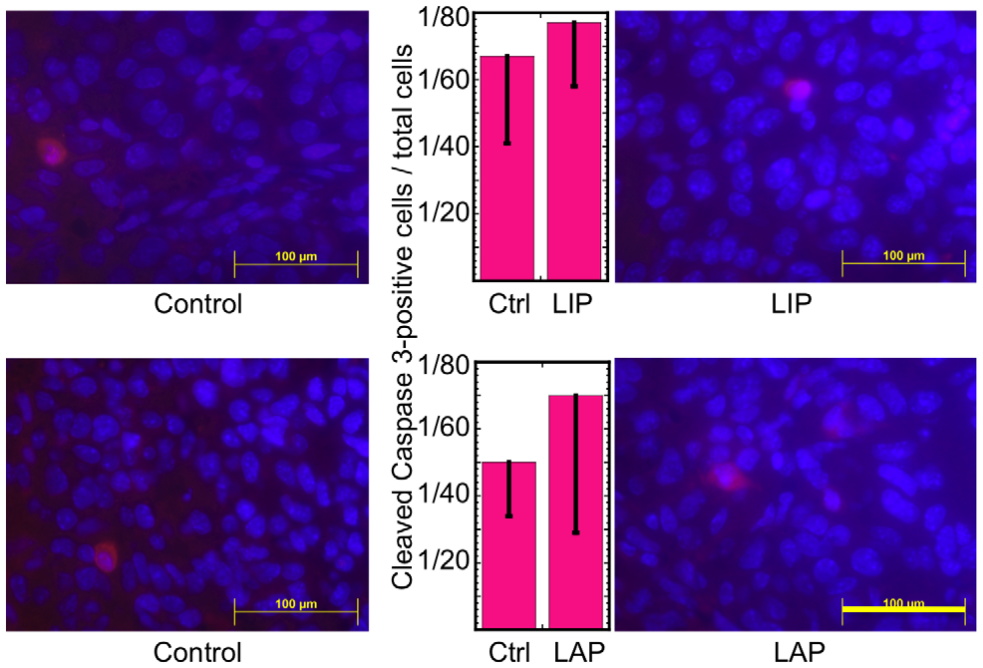
Cleaved caspase-3 staining of tumor sections. Sections of control F10.9-4 tumors and LIP-expressing F10.9-4 tumors of similar sizes, isolated at day 8 (top panels), were immunostained with an antibody against cleaved caspase 3, and nuclei were counter-stained with Hoechst 33258. The fraction of cleaved-caspase3-positive cells was determined by counting sections from 3 control and 3 LIP-expressing tumors (57–94 cells/field, middle top panel). Sections of control F10.9-3 tumors and LAP-expressing F10.9-3 tumors of similar sizes, isolated at day 8 (bottom panels), were prepared and stained as above. The fraction of cleaved-caspase3-positive cells was determined by counting sections from 3 control and 3 LAP-expressing tumors (67–109 cells/field, middle bottom panel). Representative images of sections from 3 tumors are shown. Bar = 0.1 mm.

To distinguish between the possible effects of LAP on tumor cell proliferation and cell death we stained tumor sections with the proliferation marker Ki-67 [37]. The percent of Ki67 (dividing) cells in tumors isolated at day 8 was not significantly affected by over-expression of LIP (*P* = 0.570, N = 4; **Figure 6A** **, top panels**). In accordance with our results in vitro (**Figure 3**), over-expression of LAP in the tumor resulted in a statistically significant reduction of Ki67-positive cells (*P*<0.03, N = 4; **Figure 6A**, **bottom panels**). Hence, the increase in the tumor mass upon induction of LAP must have been due to reduction in cell death, which apparently overcame the anti-proliferative effect of LAP. Indeed, necrotic areas were abundant in the tumor sections (**Figure 6B**, block arrows). Therefore, we focused our study on the impact of LAP and LIP on the extent of tumor cell death. Immunostaining of tumor sections isolated at day 8 revealed a rather low frequency (≤3%) of cleaved caspase 3-positive cells in control tumors and in tumors expressing either LAP or LIP. Furthermore, expression of either LIP or LAP had no statistically significant effect on the fraction of the cleaved caspase 3-positive cells (*P* = 0.64, N = 3 and *P* = 0.52, N = 3 for LIP and LAP, respectively; **Figure 7**). Hence, similarly to the in vitro study (**Figure 4**), spontaneous B16 melanoma cell death in vivo was probably mediated by a caspase-3 independent mechanism.

### LAP Attenuates Hypoxia–and Nutrient Deprivation–Triggered Cell Death

**Figure 8:**
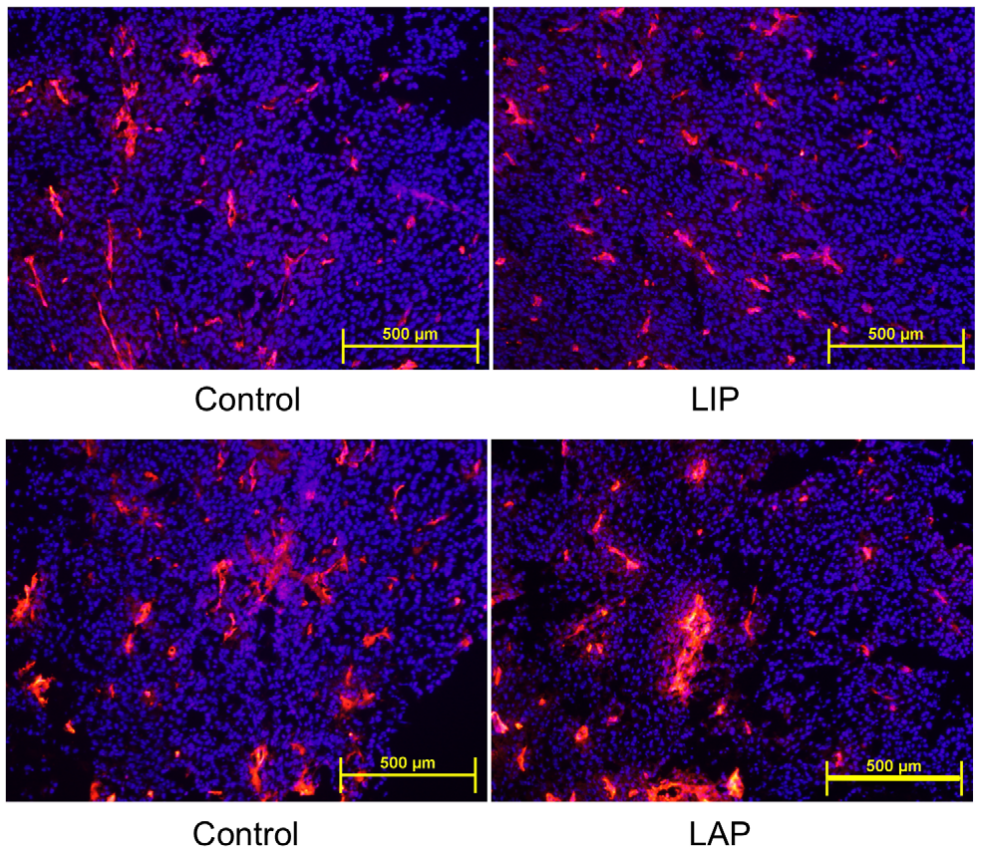
Vascularization of control and LIP–or LAP–expressing tumors. Sections of control and LIP–expressing tumors of similar sizes, isolated at day 8, (top panels) were stained with CD31 antibodies, and nuclei were counter-stained with Hoechst 33258. Sections of control and LAP–expressing tumors of similar sizes, isolated at day 8 (bottom panels), were stained with CD31 antibodies. The photographs are representatives of sections obtained from 3 tumors from each one of the four treatment groups.

**Figure 9:**
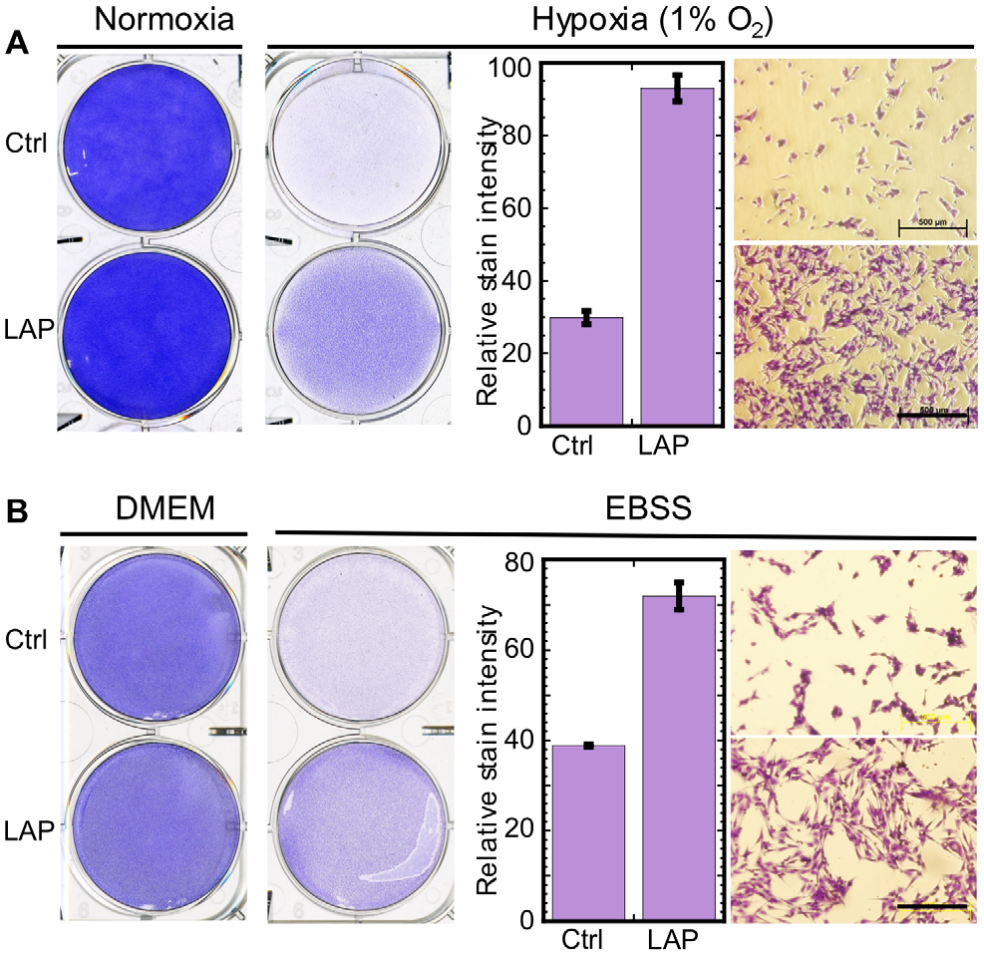
LAP attenuates hypoxia–and nutrient deprivation–triggered cell death. (A) F10.9-3 cells (4×10^5^/well) were cultured in 6-well plates in media without (Ctrl) or with (LAP) doxycycline. The plates were then cultured for 53 h, either under normoxia (8% CO_2_ in air) or under hypoxia (1% O_2_ and 8% CO_2_ in nitrogen). The plates were then stained with crystal violet and photographed (left two panels, 1x; right two panels, light microscopy. The photographs are representatives of 4 cultures. Bar = 0.5 mm. Relative cell staining intensity is shown in the middle panel. The data are average±SD of measuring four fields. (B) F10.9-3 cells (3×10^5^/well) were cultured in 6-well plates in media without (Ctrl) or with (LAP) doxycycline. The plates were then cultured either in DMEM or in serum-free EBSS for 100 h and then stained and photographed as in (A). The photographs are representatives of 4 independent cultures. Bar = 0.5 mm. Relative cell staining intensity is shown in the middle panel. The data are average±SD of measuring four fields.

LAP could attenuate tumor cell death indirectly by inducing angiogenic factors or by modulating intrinsic tumor cell functions. Staining of frozen tumor sections with antibodies to the endothelial cell marker CD31 did not reveal a significant effect of LAP on the extent of tumor vascularization (**Figure 8**). Hence, we concluded that LAP attenuated mechanisms that trigger tumor cell death or rendered the cells more resistant to such mechanisms. We therefore studied the impact of LAP on B16 cell survival upon triggering ER stress by the tumor-related stressors hypoxia and nutrient deprivation. To this end, we employed extreme hypoxic conditions (1% oxygen) or media completely lacking nutrients (serum-free Earle's Balanced Salt Solution (EBSS)) in a series of in vitro studies. As expected, hypoxia or nutrient deprivation led to massive ER stress-triggered cell death (**Figure 9**). Over-expression of LAP significantly attenuated the extent of cell death (*P*<0.0001, N = 4 for hypoxia; *P*<0.0002, N = 4 for starvation; determined by measuring the relative Crystal Violet staining intensity; **Figure 9**). These results were in accordance with the impact of LAP on the specific inducers of ER stress tunicamycin and thapsigargin.

### LAP Attenuates Tumor ER Stress

**Figure 10:**
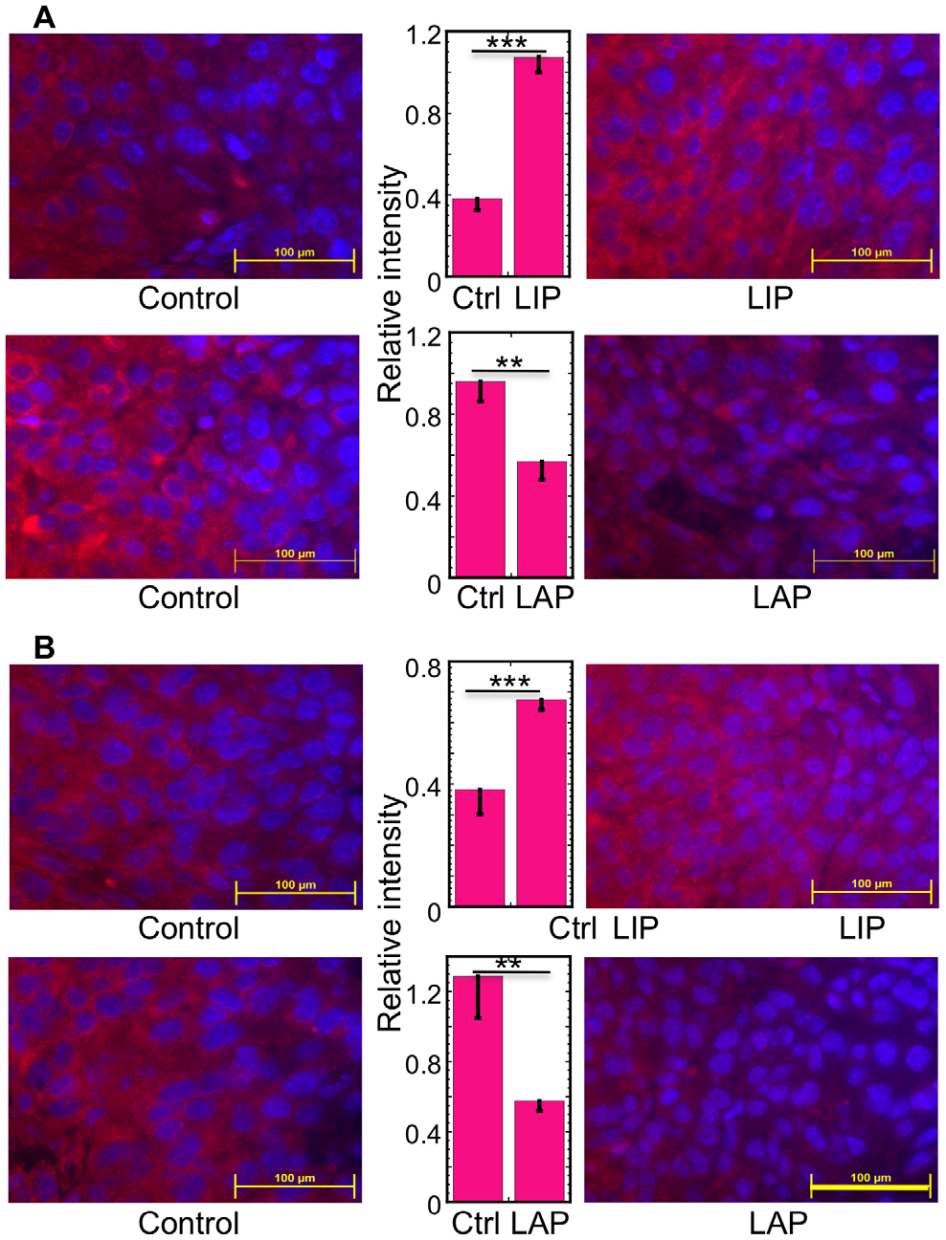
LAP attenuates tumor ER stress. (A) Sections of control F10.9-4 tumors and dominant negative LIP-expressing F10.9-4 tumors of similar sizes, isolated at day 8 (top panels), were immunostained with antibodies against the ER stress marker TRIB3. Nuclei were counter-stained with Hoechst 33258. The average staining intensity of the red channel (immunostain) was normalized to that of the blue channel (Hoechst 33258). Data are mean±SD of sections from three tumors (middle top panel). Sections of control F10.9-3 tumors and LAP-expressing F10.9-3 tumors of similar sizes, isolated at day 8 (bottom panels), were prepared, stained and analyzed as above. Data are mean±SD of sections from 5 tumors. Representative images are shown. Bar = 0.1 mm. (B) The same experiment as in (A), except that the sections were immunostained with antibodies against the ER stress marker HERPUD. Data are mean±SD of sections from 3 and 4 tumors (middle top and bottom panels, respectively). Representative images are shown. Bar = 0.1 mm.

Hypoxia, nutrient deprivation and accumulation of toxic metabolites lead to tumor cell death by triggering ER stress. Therefore, we examined the impact of LAP inhibition or over-expression on ER stress in the tumors. To this end, we immunostained sections obtained from tumors of similar size at day 8 for two ER stress markers, TRIB3 and HERPUD [38], [39]. Inhibition of endogenous LAP by over-expression of LIP significantly increased the expression of TRIB3 (*P*<0.0003, N = 3) and HERPUD (*P*<0.0005, N = 5), whereas over-expression of LAP reduced the expression of these markers (*P*<0.01, N = 3 for TRIB3; *P*<0.01, N = 4 for HERPUD) (**Figure 10**). The reduced expression of the CHOP-induced death executor TRIB3 indicated that the tumor progression and the inhibition of ER stress–triggered cell death in vitro share a common mechanism, mediated by C/EBP-β.

## Discussion

Tumor progression depends on the ability of its cells to adapt to ER stress due to lagging development of the neovasculature, which lead to nutrient limitation, impaired clearance of toxic metabolites and hypoxia. The combination of these constraints with the inherent hypermutability of cancer cells selects for cells resistant to ER stress–triggered cell death. Such resistance may be achieved by constitutive activation of the protective phase of the UPR [40], [41]. Despite significant advances in recent years [22], many features of tumor cell resistance to ER stress are unknown. The present study demonstrates the critical role of resistance to ER stress in tumor progression and shows that C/EBP-β modulates the ER stress-triggered cell death. We suggest that selection of LAP over-expressing cells, as reported in many types of cancers, enables the growing tumor to thrive despite continuous exposure to an ER stress–inducing environment. Although further studies are required to confirm the mode of action of C/EBP-β in the tumor, it is tempting to speculate that LAP or certain LAP-induced genes may serve as potential therapeutic targets for cancer and for other ER stress–mediated diseases.

The reported induction of C/EBP-β by ER stress [3] provided a general physiological framework for these studies on top of the tumor-related pathophysiology. Earlier studies with *C/EBP-β*-deficient cells and mice yielded conflicting results on the role of C/EBP-β in cell survival and death. Over-expression of LAP has been described in several cancer cell lines and was implicated in oncogenic transformation [25], [42]. Other studies found that C/EBP-β was pro-apoptotic [43], [44]. Such conflicting observations are not unexpected, as the single C/EBP-β mRNA is alternatively translated to both transcriptional activators (LAP* and LAP) and to their natural, dominant-negative inhibitor (LIP). Interactions of LAP and LIP with other transcription factors may also explain these conflicting results, as seen by us upon over-expression of LIP in HeLa cells. However, by using B16 melanoma clones that individually express either inducible LAP or LIP (**Figure S1**), we were able to determine the specific impact of LAP and LIP on the cell fate under ER stress in vitro and in tumors in vivo. Induction of LIP results in suppression of endogenous LAP activity. Induction of LAP, on the other hand, mimics the over-expression seen in many tumors.

Our finding that LAP attenuated whereas LIP augmented ER stress–triggered cell death in vitro (**Figures 1** **,** **2** **&S2**) correlated with the observed fourfold increase in the size of LAP-expressing tumors during days 8-13 and the mirror image fourfold decrease in size of LIP-expressing tumors (**Figure 5**). The protective effect exerted by LAP in cells exposed to nutrient deprivation or hypoxia further supports this correlation (**Figure 9**). The inhibition of the expression of death executor CHOP by LAP under ER stress (**Figure 4**) provides one mechanism by which LAP attenuated ER stress-triggered cell death in vitro. Since the death executor TRIB3 is induced by CHOP, our finding that LAP attenuated and LIP augmented the expression of TRIB3 in the tumor (**Figure 10A**) suggests that LAP promotes tumor progression by attenuating CHOP-mediated cell death. By performing knockdown and over-expression studies in two other human cell types (**Figure S4**) we demonstrated the general role of LAP and LIP in modulating the resistance to ER stress-triggered cell death. A previous study demonstrated that C/EBP-β null mice (lacking both LAP and LIP) are completely refractory to generation of skin tumors upon exposure to a carcinogen [8]. Our findings further show that C/EBP-β promotes the progression of an established tumor by attenuating cell death, implicating ER stress in the process.

Our findings suggest that changes in the LAP-LIP ratio control the transition from the early, protective UPR to its late, death–promoting phase. Others [18] and we observed that ER stress initially triggered the expression of LAP, whereas extended ER stress led to expression of both LAP and LIP (**Figure 1**). C/EBP-β was found to be constitutively bound to the CHOP promoter at the common C/EBP-ATF4 site, inhibiting *CHOP* expression in rat cells [45]. Our findings that LAP attenuated the expression of CHOP and its downstream target TRIB3 (**Figures 4C** **&** **10A**) suggest that LAP rather than LIP is the constitutive transcriptional inhibitor of *CHOP*. Thus, LIP may relieve the transcription inhibition of LAP by forming a futile LAP-LIP heterodimer and thereby triggering cell death. Alternatively or additionally, such a LAP-LIP heterodimer may trigger cell death in a manner analogous to the proposed action of the LAP-CHOP heterodimer [5]. Regardless of the exact mechanism, this study demonstrates that LAP inhibits the death-promoting phase of the UPR whereas in most cases LIP promotes ER stress–triggered cell death. Establishing the impact of LAP and LIP on gene expression under ER stress will further clarify the mechanism by which LAP attenuates cell death.

## Materials and Methods

### Ethics Statement

The Weizmann Institute Animal Care and Use Committee approved all studies in mice, according to institutional guidelines and Israeli law.

### Cells and Reagents

The murine B16 melanoma clones, F10.9-3 and F10.9-4, were derived from the parental cell line B16-F10 (ATCC CRL-6475) and were kindly provided by M. Revel and J. Chebath (Weizmann Institute of Science) [34]. Human cervical carcinoma HeLa cells (CCL-2.1) and human embryonic kidney cells (HEK 293T) were obtained from the American Type Culture Collection (ATCC), Manassas, VA. Cell culture media were supplemented with 10% FBS and antibiotics (DMEM-10 and MEM-10) and cells were grown in humidified 8% CO_2_ incubators at 37°C. Expression of LAP and LIP was induced in the F10.9-3 and F10.9-4 cells, respectively by doxycycline (Doxy, 1 µg/ml, 24 h) unless otherwise stated. ER stress was elicited by treatment with tunicamycin (0.75 µg/ml, 24 h) or thapsigargin (50 nM, 48 h) unless otherwise stated. Antibodies to C/EBP-β (C-19) and CHOP (B-3) were purchased from Santa Cruz Biotechnology (Santa Cruz, CA); the TRIB3 antibody (ST1032) was from Calbiochem (San Diego, CA); the Ki-67 antibody (clone TEC-3) was from DakoCytomation (Copenhagen, Denmark); the cleaved caspase 3 antibody (Asp 175) was from Cell Signaling (Danvers, MA); the HERPUD antibody was from Protein Tech Group (Chicago, IL), the rat anti-mouse CD31 (PECAM-1) and BiP/GRP78 antibodies were from BD Pharmingen, (San Diego, CA), the HMGB1 antibody (H9539) was from Sigma-Aldrich (Rehovot, Israel) and the Cy3-conjugated streptavidin, biotin-SP–conjugated AffiniPure donkey anti-rabbit IgG and biotin-SP–conjugated goat anti-mouse IgG antibodies were from Jackson ImmunoResearch (Baltimore, PA). All other reagents were purchased from Sigma-Aldrich (Rehovot, Israel).

### Tumor Generation and Immunohistochemistry

C57/BL6 mice were inoculated subcutaneously with F10.9-3 or F10.9-4 cells (100 µl medium, containing 5×10^5^ cells mixed with 100 µl Matrigel). Doxycycline (1 mg/ml) was added to the drinking water for the induction of LAP and LIP. Mice (10 per group) were euthanized at day 8 or 13, and tumors were resected, weighed, photographed and either fixed in 10% formaldehyde or frozen before sections were prepared. The significance of differences in the tumor mass was determined by Student's t-test of independent samples. The paraffin sections were immunostained with antibodies to Ki-67, cleaved caspase 3, TRIB3 and HERPUD, followed by incubation with biotin-conjugated antibodies and Cy3-conjugated streptavidin. Cell nuclei in tumor slices and in cultures were stained with Hoechst 33258 (5 µg/ml; excitation at 365 nm and emission at 470 nm). For CD31 immunostaining, tumor tissues were embedded in OCT and were frozen at −80°C for cryosectioning. After fixation in 100% acetone (−20°C, 20 min), slides were immunostained as described above.

### Quantitative Measurment of the Immunostains

The percentage of proliferating cells was determined by counting ki67-positive nuclei and dividing by the total number of (Hoechst 33258-stained) nuclei. The fraction of cleaved caspase 3 cells was similarly determined. The staining intensity of TRIB3 and HERPUD was determined by the histogram function of The Photoshop program (red channel) and normalized to the staining intensity of the nuclei (blue channel) in the same field. The significance of differences in cell counts or in the staining intensity was determined by Student's t-test on the indicated number of replicates.

### RNAi–Mediated Knockdown

siRNA pools (ON-TARGETplus), directed against murine and human C/EBP-β mRNA (Genebank accession No. NM_009883 and NM_005194, respectively) were purchased from Dharmacon RNAi Technologies (Lafayette, CO). The knockdown of the transcripts was performed in 6-well plates with DharmaFECT 1 reagent, according to the manufacturer's protocol. Cells were seeded in media without antibiotics for the knockdown experiments.

### Cell Staining, Fluorescence Microscopy and Flow Cytometry

To evaluate the extent of cell death, cultures were stained first with Hoechst 33258 (5 µg/ml, 15 min) washed and stained with propidium iodide (2.5 µg/ml, 15 min) and then visualized by fluorescence microscopy. To evaluate cell morphology, cultures were fixed and stained with 5% crystal violet in 66% methanol. Total cell counts were determined by counting manually, by using the ImageJ program (NIH) or by reading the crystal violet stained cultures in 96 well plates with an ELISA reader at 570 nm. Alternatively, staining intensity was determined in photographs by the histogram function of Photoshop. For quantitative measurement of viable cells, cultures in 96 well plates were incubated with Neutral Red (70 mg/L in 0.1 ml DMEM-10, 37°C, 30–60 min), washed 3X with PBS, re-suspended in lysis buffer (0.1 ml) and OD was measured with an ELISA reader at 540 nm. A calibration curve was prepared by seeding known amounts of cells onto 96-well plates. After 6 h the cells were stained with Neutral Red and the OD was read as described above. For each experiment, statistical analyses were performed on the indicated number of independent experiments using Student's *t*-test of the independent samples. Each independent experiment consisted of 9 replicates. Apoptosis was evaluated by staining with the rh annexin-V/FITC kit (Bender MedSystems, Vienna, Austria). Cell cycle analysis was performed by collecting the cultured cells, fixing in 70% ethanol on ice, treatment with RNase A (100 µg/ml, 30 min, 37°C), staining with propidium iodide and flow cytometry.

### Immunoblotting of Cellular Proteins

Cells were washed three times with ice-cold phosphate buffered saline (PBS). Cell pellets were resuspended in two packed cell volume of lysis buffer (20 mM Hepes, pH 7.9, 0.42 M NaCl, 1.5 mM MgCl2, 0.2 mM EDTA, 0.5 mM Phenyl Methyl Sulfonyl Flouride (PMSF), 0.5 mM dithiothreitol (DTT), 25% glycerol). The resuspended pellets were then frozen and thawed twice and kept on ice for 20 min. The lysates were centrifuged (14,000 rpm, 20 min.) and the supernatants containing the cellular proteins were collected, frozen in liquid nitrogen and stored at −80°C. Protein concentration was determined by a BCA Protein assay reagent kit (Pierce, Rockford USA) using bovine serum albumin as a standard. Protein samples were boiled in SDS-PAGE sample buffer containing 25 mM DTT, and the supernatants were resolved by gradient SDS-PAGE (7.5–15% acrylamide). Proteins were then transferred onto a nitrocellulose membrane, which was incubated with the indicated antibodies. Second antibody conjugates were visualized by the Super Signal Detection Kit (Pierce).

### Construction of pcLAP and pcLIP Expression Vectors

To construct a mammalian expression vector that expresses only LAP (pcLAP), the complete human C/EBP-β ORF was inserted into pcDNA4 (Life Technologies, Carlsbad CA). Codon 1 (LAP*-initiating ATG) was point-mutated to CTG using Pfu Turbo DNA polymerase (Stratagene, La Jolla, CA), the forward oligo 5′ TGTGCTGGAATTCC**C**TGCAACGCCTGGTGG and the reverse oligo 5′ ACGGGAAGCCCGCCGCCA**G**GCCTGCGCCGCCGC. Codon 199 (ATG) was then point-mutated as above with the forward oligo 5′ GCGGCGGCGCAGGC**C**TGGCGGCGGGCTTCCCGT and the reverse oligo 5′ ACGGGAAGCCCGCCGCCA**G**GCCTGCGCCGCCGC to prevent expression of LIP. The LIP ORF (codons 199–346) was inserted into pcDNA4 to generate pcLIP, which expresses only the human LIP isoform of C/EBP-β.

## Supporting Information

Figure S1 Inducible over-expression of LAP and LIP in murine B16 F10.9 clones. B16 F10.9 cells (4×10^5^/well) were cultured in 6-well plates with or without doxycycline (Doxy). (A) RT-PCR of C/EBP-β mRNA from F10-9.3 cells. (B) GFP expression in F10 9.3 cells. Bar = 0.5 mm. (C) Dose response of LAP expression in F10 9.3 cells following 24 h treatment with increasing concentrations of doxycycline, as determined by immunoblotting. (D) Dose response of LIP expression in F10 9.4 cells, as described in (C). The gels and blots are representative results of three replicate experiments.
(1.00 MB TIF)

Figure S2 LIP augments and LAP attenuates ER stress-triggered cell death in a dose-dependent manner. (A) F10.9-4 cells (2×10^4^/well) were cultured in 96-well plates with the indicated concentration of doxycycline for 24 h. Diluent (Ctrl), tunicamycin (Tm) or thapsigargin (Tg) were then added. After an additional 24 h the plates were stained with crystal violet and photographed. The photograph is representative of five replicate experiments. (B) F10.9-3 cells (1.5×10^4^/well) were cultured in 96-well plates with the indicated concentration of doxycycline for 24 h. Diluent (Ctrl), tunicamycin (Tm) or thapsigargin (Tg) was then added. After 24 h the plates were stained with crystal violet and photographed under a light microscope. Control cells were also photographed by fluorescent microscopy (top panel) to evaluate the extent of GFP and C/EBP-β induction by doxycycline. The photograph is a representative of five replicate experiments. Bar = 0.5 mm.
(9.92 MB TIF)

Figure S3 Survival and death of F10.9-3 cells under ER stress. (A) F10.9-3 cells were seeded in 8 well microscopy chambers (Integrated Biodiagnostics, Munich, Germany; 40,000/well) and after 24 h challenged either with tunicamycin (0.5 µg/ml, 24 h) or with TNF-α (100 ng/ml) plus IFN-γ (1000 IU/ml) for 24 h. The cells were stained for apoptosis by the rh annexin-V/FITC kit followed by Hoechst 33258. The treatment with TNF-α plus IFN-γ served as a positive control for apoptosis, as determined by the annexin V/FITC staining. The photographs are representatives of four replicate studies. Bars are 0.2 mm. Insets: larger magnification to show cellular staining details. Bar = 0.05 mm. (B) F10.9-3 cells in 6-well plates were challenged with tunicamycin (1 µg/ml, 48 h). The detached cells were collected, plated and stained with propidium iodide and counter-stained with Hoechst 33258. The photograph is a representative of three replicate experiments. Bar = 0.2 mm. (C) Control (Ctrl) and doxycycline-treated F10.9-3 cells (LAP) were challenged with tunicamycin (Tm) or Thapsigargin (Tg) as described in Figure 2B. The cells that remained attached to the plates were stained with crystal violet and photographed. The photographs are representatives of five replicate experiments. Bar = 0.2 mm.
(4.02 MB TIF)

Figure S4 Effects of LAP and LIP knockdown or over-expression on cell survival upon ER stress. (A) Human 293T cells in 6 well plates were transfected with 1 µg of control pcDNA4 (middle lane), pcLAP (left lane), or pcLIP (right lane). After 24 h cell extracts (20 µg protein) were immunoblotted with antibodies directed to the common C-terminus of C/EBP-β. N.S. is non-specific bands. The blot is a representative of three replicates. (B) Human 293T cells were transfected as in E and after 24 h were trypsin-digested and seeded in poly(D-lysine)-coated 96 well plates (20,000 cells/well). After additional 24 h the cells were either untreated (Ctrl) or challenged with tunicamycin (2 µg/ml). The plates were left for 24 h and the number of viable cells was determined by the Neutral Red assay. Percent survival represent the ratio of tunicamicyn-treated to diluent-treated cells. Data are mean±SD of four replicates. Similar transfection efficiencies were obtained with the three vectors and cell counts were very similar in the control cultures not treated with tunicamycin (Ctrl). (C) HeLa cells (2×10^5^/well) were cultured in 6-well plates for 24 h and then transfected with a control siRNA or C/EBP-β-specific siRNA. After 24 h, RNA was isolated and analyzed by RT-PCR (left panels), and cell extracts were prepared and analyzed by immunoblotting with antibodies directed to the common C-terminus of C/EBP-β (right panels). The gels and blots are representatives of three replicate experiments. (D) HeLa cells were seeded and transfected as described in (C). After 36 h, diluent (Ctrl) or Tunicamycin (Tm) were added and after an additional 31 h the cultures were stained with Crystal Violet and photographed. The photographs are representatives of four replicate experiments. Bar = 0.5 mm. (E) Cell counts following the treatments described in (D). The data are average of counting four fields. (F) Human HeLa cells (200,000) were transfected with 1 µg of either control pcDNA4 vector, pcLAP vector (mutated to encode LAP only), or pcLIP vector (encoding LIP only). After 24 h the cells were seeded in 96 well plates (20,000 cells/well) and after additional 24 h diluent or tunicamycin (4 µg/ml) were added. After 48 h cell viability was determined by Neutral Red staining. Percent viability was determined by the ratio of tunicamycin to diluent-treated cells. Data are mean±SD of six replicates.
(2.03 MB TIF)
