# Correction: C/EBP-β Regulates Endoplasmic Reticulum Stress–Triggered Cell Death in Mouse and Human Models

**DOI:** 10.1371/annotation/af6dfc34-7211-4e27-be19-298f08ec33f6

**Published:** 2010-03-23

**Authors:** Ofir Meir, Efrat Dvash, Ariel Werman, Menachem Rubinstein

There is an error with the beta symbols in the abstract. The abstract should read: Endoplasmic reticulum (ER) stress elicits the unfolded protein response (UPR), initially aimed at coping with the stress, but triggering cell death upon further stress. ER stress induces the C/EBP-β variant Liver-enriched Activating Protein (LAP), followed by the dominant-negative variant, Liver Inhibitory Protein (LIP). However, the distinct role of LAP and LIP in ER stress is unknown. We found that the kinetics of the ER stress-induced expression of LIP overlapped with that of the cell death in mouse B16 melanoma cells. Furthermore, inducible over-expression of LIP augmented ER stress-triggered cell death whereas over-expression of LAP attenuated cell death. Similar results were obtained in human 293T cells. Limited vasculature in tumors triggers hypoxia, nutrient shortage and accumulation of toxic metabolites, all of which eliciting continuous ER stress. We found that LAP promoted and LIP inhibited B16 melanoma tumor progression without affecting angiogenesis or accelerating the cell cycle. Rather, LAP attenuated, whereas LIP augmented tumor ER stress. We therefore suggest that C/EBP-β regulates the transition from the protective to the death-promoting phase of the UPR. We further suggest that the over-expression of LAP observed in many solid tumors promotes tumor progression by attenuating ER stress-triggered tumor cell death.

