# Supplementary figures and images for: C/EBP-β Regulates Endoplasmic Reticulum Stress–Triggered Cell Death in Mouse and Human Models
Source: PLoS One. 2010 Mar 3;5(3):e9516. doi: 10.1371/journal.pone.0009516 (PMC2831074; doi:10.1371/journal.pone.0009516)

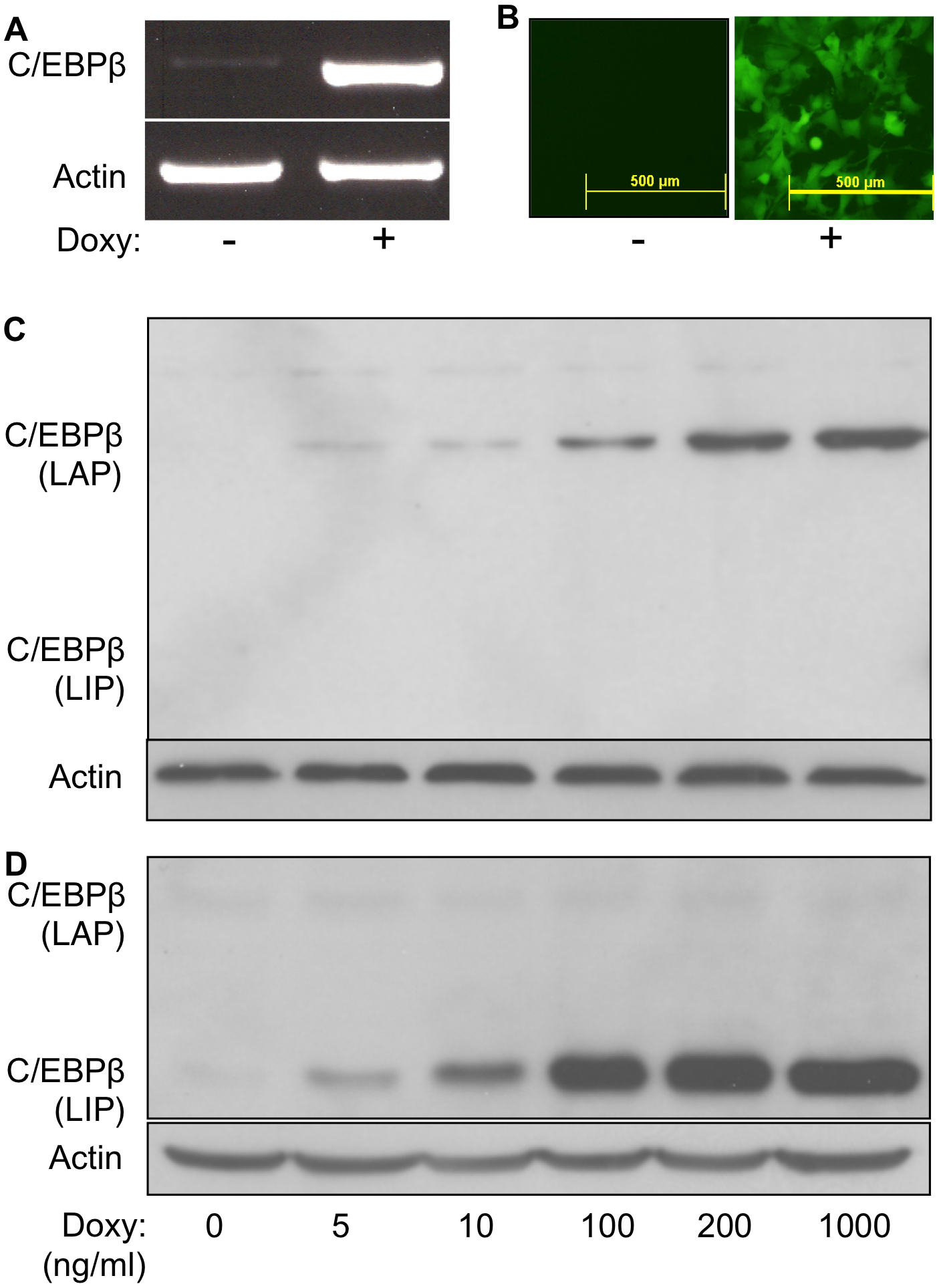

Supplement: Figure S1 — Inducible over-expression of LAP and LIP in murine B16 F10.9 clones. B16 F10.9 cells (4×10^5^/well) were cultured in 6-well plates with or without doxycycline (Doxy). (A) RT-PCR of C/EBP-β mRNA from F10-9.3 cells. (B) GFP expression in F10 9.3 cells. Bar = 0.5 mm. (C) Dose response of LAP expression in F10 9.3 cells following 24 h treatment with increasing concentrations of doxycycline, as determined by immunoblotting. (D) Dose response of LIP expression in F10 9.4 cells, as described in (C). The gels and blots are representative results of three replicate experiments. (1.00 MB TIF) [file pone.0009516.s001.tif]

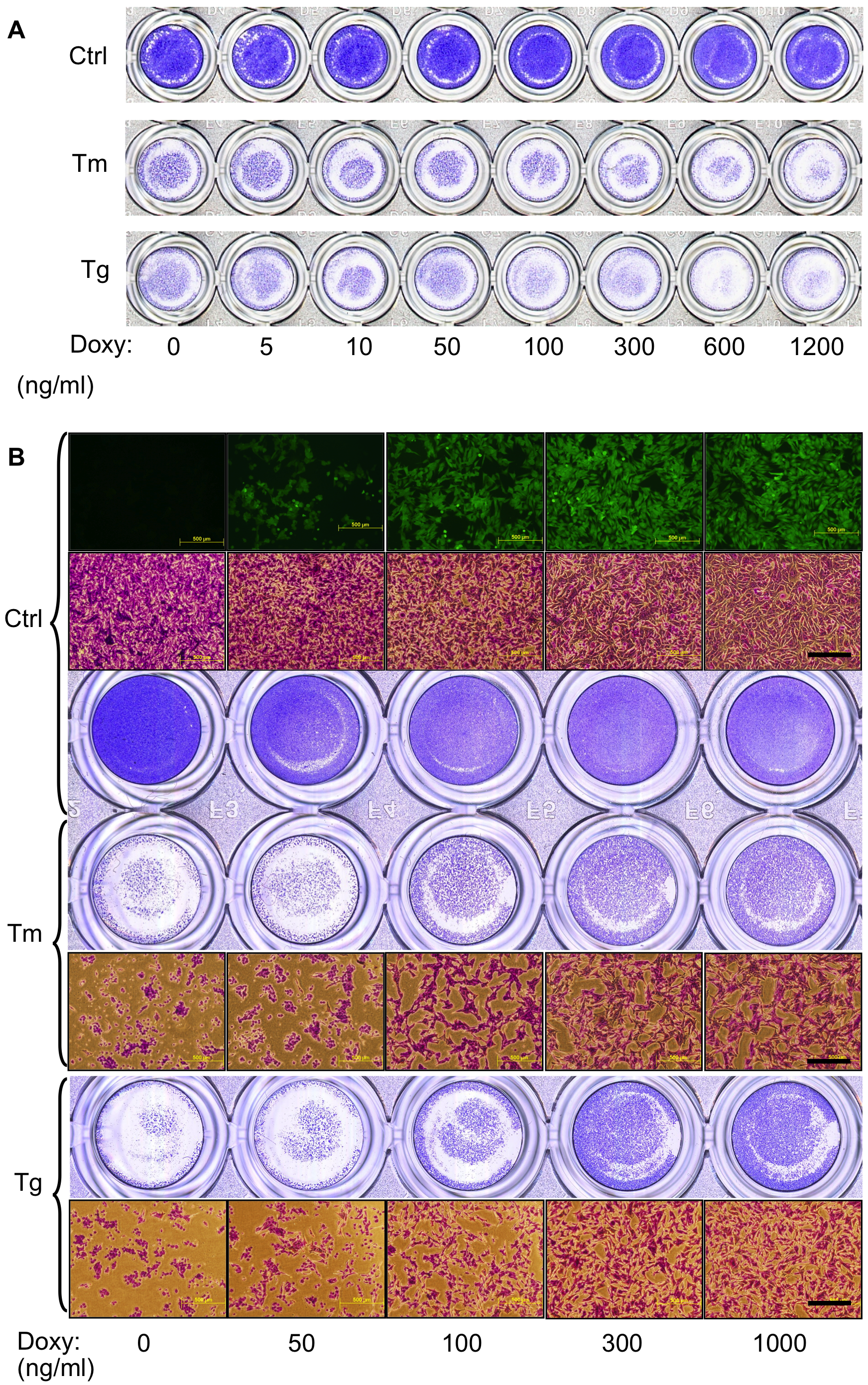

Supplement: Figure S2 — LIP augments and LAP attenuates ER stress-triggered cell death in a dose-dependent manner. (A) F10.9-4 cells (2×10^4^/well) were cultured in 96-well plates with the indicated concentration of doxycycline for 24 h. Diluent (Ctrl), tunicamycin (Tm) or thapsigargin (Tg) were then added. After an additional 24 h the plates were stained with crystal violet and photographed. The photograph is representative of five replicate experiments. (B) F10.9-3 cells (1.5×10^4^/well) were cultured in 96-well plates with the indicated concentration of doxycycline for 24 h. Diluent (Ctrl), tunicamycin (Tm) or thapsigargin (Tg) was then added. After 24 h the plates were stained with crystal violet and photographed under a light microscope. Control cells were also photographed by fluorescent microscopy (top panel) to evaluate the extent of GFP and C/EBP-β induction by doxycycline. The photograph is a representative of five replicate experiments. Bar = 0.5 mm. (9.92 MB TIF) [file pone.0009516.s002.tif]

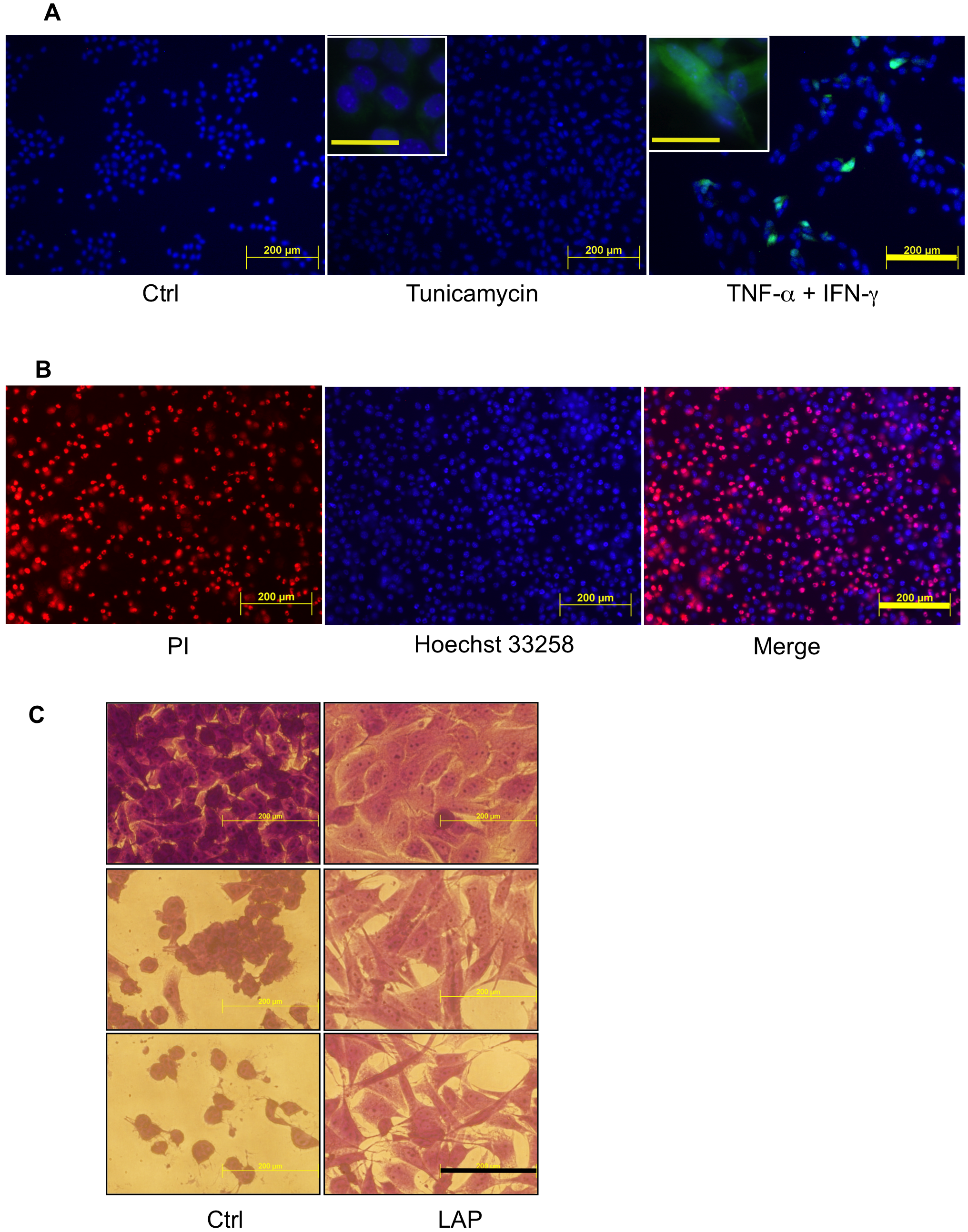

Supplement: Figure S3 — Survival and death of F10.9-3 cells under ER stress. (A) F10.9-3 cells were seeded in 8 well microscopy chambers (Integrated Biodiagnostics, Munich, Germany; 40,000/well) and after 24 h challenged either with tunicamycin (0.5 µg/ml, 24 h) or with TNF-α (100 ng/ml) plus IFN-γ (1000 IU/ml) for 24 h. The cells were stained for apoptosis by the rh annexin-V/FITC kit followed by Hoechst 33258. The treatment with TNF-α plus IFN-γ served as a positive control for apoptosis, as determined by the annexin V/FITC staining. The photographs are representatives of four replicate studies. Bars are 0.2 mm. Insets: larger magnification to show cellular staining details. Bar = 0.05 mm. (B) F10.9-3 cells in 6-well plates were challenged with tunicamycin (1 µg/ml, 48 h). The detached cells were collected, plated and stained with propidium iodide and counter-stained with Hoechst 33258. The photograph is a representative of three replicate experiments. Bar = 0.2 mm. (C) Control (Ctrl) and doxycycline-treated F10.9-3 cells (LAP) were challenged with tunicamycin (Tm) or Thapsigargin (Tg) as described in Figure 2B. The cells that remained attached to the plates were stained with crystal violet and photographed. The photographs are representatives of five replicate experiments. Bar = 0.2 mm. (4.02 MB TIF) [file pone.0009516.s003.tif]

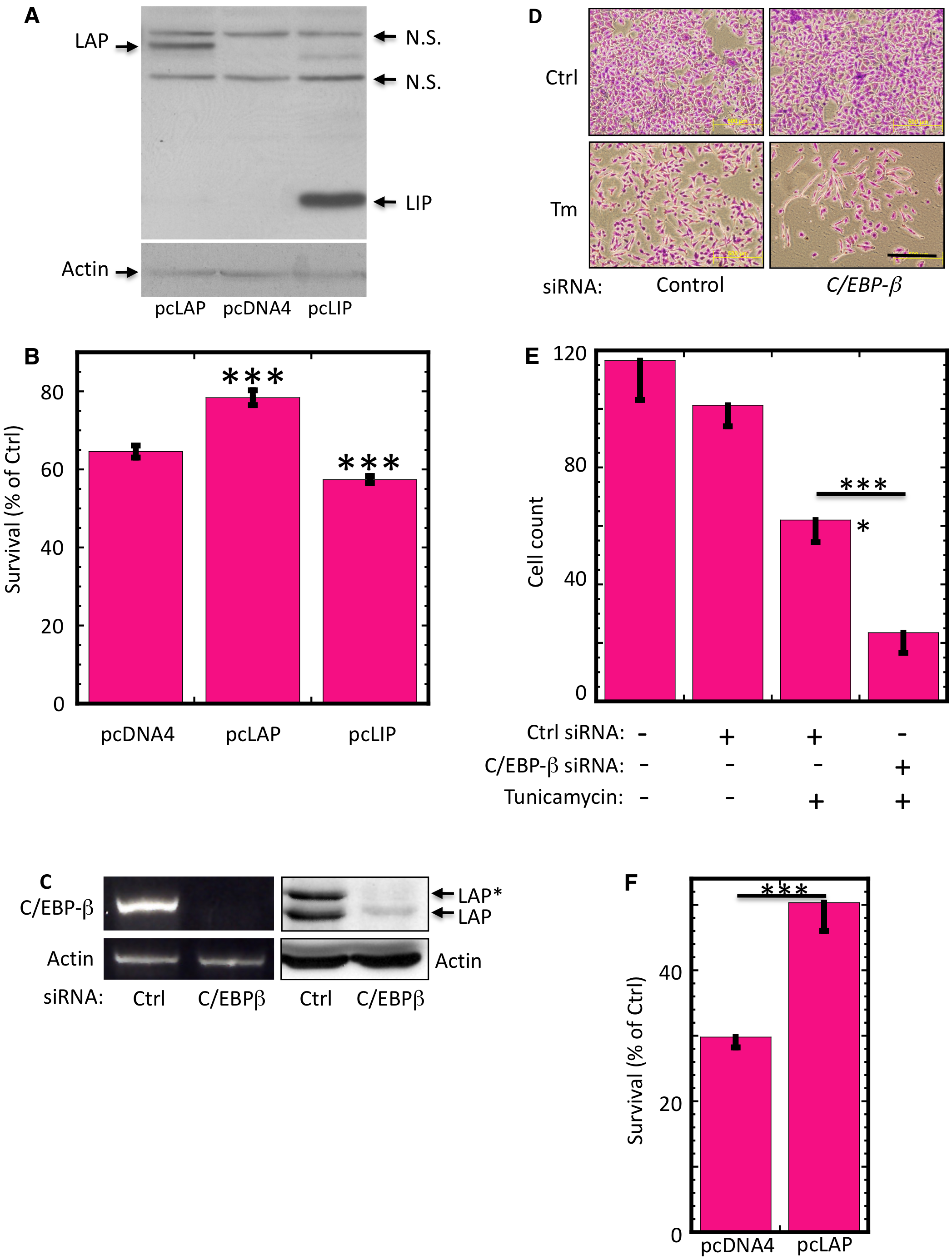

Supplement: Figure S4 — Effects of LAP and LIP knockdown or over-expression on cell survival upon ER stress. (A) Human 293T cells in 6 well plates were transfected with 1 µg of control pcDNA4 (middle lane), pcLAP (left lane), or pcLIP (right lane). After 24 h cell extracts (20 µg protein) were immunoblotted with antibodies directed to the common C-terminus of C/EBP-β. N.S. is non-specific bands. The blot is a representative of three replicates. (B) Human 293T cells were transfected as in E and after 24 h were trypsin-digested and seeded in poly(D-lysine)-coated 96 well plates (20,000 cells/well). After additional 24 h the cells were either untreated (Ctrl) or challenged with tunicamycin (2 µg/ml). The plates were left for 24 h and the number of viable cells was determined by the Neutral Red assay. Percent survival represent the ratio of tunicamicyn-treated to diluent-treated cells. Data are mean±SD of four replicates. Similar transfection efficiencies were obtained with the three vectors and cell counts were very similar in the control cultures not treated with tunicamycin (Ctrl). (C) HeLa cells (2×10^5^/well) were cultured in 6-well plates for 24 h and then transfected with a control siRNA or C/EBP-β-specific siRNA. After 24 h, RNA was isolated and analyzed by RT-PCR (left panels), and cell extracts were prepared and analyzed by immunoblotting with antibodies directed to the common C-terminus of C/EBP-β (right panels). The gels and blots are representatives of three replicate experiments. (D) HeLa cells were seeded and transfected as described in (C). After 36 h, diluent (Ctrl) or Tunicamycin (Tm) were added and after an additional 31 h the cultures were stained with Crystal Violet and photographed. The photographs are representatives of four replicate experiments. Bar = 0.5 mm. (E) Cell counts following the treatments described in (D). The data are average of counting four fields. (F) Human HeLa cells (200,000) were transfected with 1 µg of either control pcDNA4 vector, pcLAP vector (mutat [file pone.0009516.s004.tif]
